# Tailoring services in opioid treatment programs for patients involved in America’s criminal justice system: national associations and variation by state and Medicaid expansion status

**DOI:** 10.1186/s13011-021-00388-5

**Published:** 2021-06-19

**Authors:** George Pro, Brooke E. E. Montgomery, Nickolas Zaller

**Affiliations:** Southern Public Health and Criminal Justice Research Center and the Department of Health Behavior and Health Education in the Fay W. Boozman College of Public Health, University of Arkansas for Medical Sciences, Little Rock, USA

**Keywords:** Opioid use disorder, Opioid treatment programs, Criminal justice, Epidemiology

## Abstract

**Background:**

Opioid treatment programs (OTPs) are the primary source of medication-assisted treatment (MAT) for many individuals with opioid use disorder, including poor and uninsured patients and those involved in the criminal justice (CJ) system. Substance use treatment services that are tailored to the unique needs of patients often produce better outcomes, but little national research has addressed characteristics associated with whether OTPs offer services specifically tailored to community members involved in the CJ system. Medicaid expansion under the Affordable Care Act has broadly strengthened MAT services, but the role of expansion in supporting MAT services that are specifically tailored towards CJ-involved populations remains unknown. Moreover, it is unknown whether the availability of tailored services varies between Medicaid expansion states.

**Methods:**

We used the 2019 National Survey of Substance Abuse Treatment Services to identify OTPs in the US (*n* = 1679) and whether they offered services specifically tailored for CJ-involved patients. We used logistic regression to model the association between OTPs offering tailored services and state Medicaid expansion status, adjusted for state-level opioid overdose and community supervision rates.

**Results:**

Nationally, only a quarter of OTPs offered services tailored to CJ populations, and the majority of OTPs (73%) were located in Medicaid expansion states. Compared to OTPs in non-expansion states, OTPs in expansion states demonstrated nearly double the odds of offering tailored services (adjusted odds ratio = 1.90, 95% confidence interval = 1.41–2.57, *p* < 0.0001). The predicted probability of offering tailored services varied by state; probability estimates for all expansion states were above the national mean, and estimates for all non-expansion states were below the national mean.

**Conclusion:**

Our findings reiterate the role of Medicaid in promoting the adoption of comprehensive OTP services for CJ-involved populations. However, the proportion of OTPs that offered tailored services was relatively low, pointing to the need to continually strengthen Medicaid services and coverage.

## Introduction

Opioid use disorders (OUD) and preventable overdose-related deaths continue to drive an ongoing national epidemic. Rates of opioid misuse have risen consistently through 2019, with the majority of overdose-related deaths attributed to fentanyl, including fentanyl mixed with heroin, other opioids, and stimulants [1]. An additional and notable uptick in misuse and overdose has also been identified during the COVID-19 pandemic [2]. Medication-assisted treatment (MAT) for OUDs is effective at reducing opioid misuse and overdose [3], but treatment uptake remains low [4]. Opioid treatment programs (OTPs) are federally licensed facilities that dispense agonist MAT medications (i.e. methadone and buprenorphine). Between 2003 and 2016, the number of OTPs in the US increased by nearly 40% [5]. Private office-based treatment is also increasingly available [6], but OTPs have historically been the primary treatment resource for OUD treatment in most of the US [7]. Despite the number of private office-based buprenorphine prescriptions recently surpassing prescriptions dispensed by OTPs [5], office-based buprenorphine is more likely to be prescribed to White clients, as well as those paying with cash or private insurance [8]. As a result, OTPs remain the primary source of MAT for disadvantaged populations, including people of color and individuals involved in the criminal justice (CJ) system.

There are disproportionate burdens of OUD, treatment barriers, and poor treatment outcomes among individuals in the community who are involved in the CJ system. The majority of CJ-involved individuals are not incarcerated; more than four million Americans are on probation, parole, or are otherwise experiencing some form of correctional supervision by a court [9]. Rates of OUD are substantially higher among CJ-involved individuals compared to their non-involved counterparts, and rates of relapse, overdose, and death are extremely high following release from prison [10]. Lower MAT uptake is related to the inability to pay for services and having limited or no health insurance [11], which are common barriers among CJ-involved populations [12–14]. In addition, Krawczyk and colleagues [15] found that individuals with OUD who were referred to community-based treatment by the CJ system (i.e., courts, probation/parole, diversion) were less likely than their non-CJ-referred counterparts to receive MAT. To highlight the scope of the public health crisis, a 2017 report by the Substance Abuse and Mental Health Services Administration (SAMHSA) [16] showed that 34% of all treatment admissions to publicly funded treatment facilities – or 682,000 people in 1 year – were there for heroin or other opioids. Of those, 94,000 (14%) were referred to treatment by the CJ system. The elevated risk of not receiving MAT showcases the additional challenges in maintaining recovery from OUD among those navigating both the CJ and treatment systems. Critically, CJ-involved community members who need but do not receive MAT are more likely to be re-arrested, have probation revoked, and be re-incarcerated [17].

Given the persistent barriers to initiating and maintaining MAT, there is a need for OTPs to better tailor services to meet the needs of CJ-involved clients. OTPs offer a reliable resource for those who would otherwise not attend private office-based treatment, but additional resources and funding for OTPs could improve maintenance on MAT and other health outcomes. Several strategies could be incorporated into OTPs to tailor services for CJ clients, including connection to MAT immediately upon release [18] and expanding telemedicine services for more rural CJ clients [19]. Reichert and Gleicher [20] found that probation department leaders and staff in Illinois had difficulty in offering guidance about MAT to probationers, largely due to a lack of understanding about the use of MAT, how it is administered, and a generally low level of familiarity with OUD and behavioral health. These findings highlight a unique opportunity for OTPs to tailor MAT services by communicating directly with probation officers about what to expect from a probationer who is receiving MAT. This linkage between MAT providers and CJ agencies is one of several critical steps in the continuum of care for individuals under community supervision [21]. Broadly, services tailored towards CJ clients should serve to improve retention and health and decrease the risks of further involvement with the CJ system and recidivism.

State Medicaid expansions under the Affordable Care Act have broadly increased access to substance use treatment services among poor and marginalized populations, including CJ-involved populations who have historically been un- and under-insured [14, 22, 23]. Increased enrollment in insurance plans in general – and Medicaid specifically – is associated with a notable uptick in health services use among CJ-involved individuals [14], and this increase in the ability to pay for services may be one motivating factor for OTPs to broaden the types of tailored services offered. In this sense, Medicaid expansion may not only improve MAT access but may also serve to strengthen existing OTP services to reach a larger and more diverse clientele. MAT prescriptions increased markedly in states that expanded Medicaid, while making only modest or no gains in states that opted out of expansions [24–27]. Most expansion states saw significant increases in MAT prescriptions without a corresponding increase in prescriptions for opioid pain relievers [28], simultaneously strengthening treatment services and decreasing the supply of opioids that could be diverted to illicit sales. Medicaid expansion has strengthened health services access for CJ-involved individuals, but results from one study by Fry and colleagues [29] that looked at the effect of expansions on CJ-specific outcomes like recidivism were mixed. However, Wen and colleagues [30] attributed reductions in the rates of robbery, aggravated assault, and larceny theft in expansion states to increases in substance use treatment. Medicaid expansion also decrease overall state spending because it is partially offset by savings made in the CJ system [31, 32].

While Medicaid expansions have generally benefited the most vulnerable populations, to our knowledge no research has looked at differences in OTP CJ-tailored services between Medicaid expansion and non-expansion states. We hypothesized that OTPs in expansion states would be more likely than OTPs in non-expansion states to offer tailored services to CJ populations. In addition, we investigated differences between individual states within each of the two expansion groups.

## Materials and methods

### Data source and variables

We used the National Survey of Substance Abuse Treatment Services (N-SSATS; 2019) [33] to identify OTPs in the US (*n* = 1679). In short, N-SSATS is planned, directed, and maintained jointly by the Center for Behavioral Health Statistics and Quality, SAMHSA, and the U.S. Department of Health and Human Services. The dataset provides annual facility-level administrative data on the state, organization, structure, and a range of clinical and ancillary services provided by both public and private substance use treatment facilities, including OTPs. No client-level information is collected or included in the dataset. In 2019, the survey response rate of facilities that provided treatment for substance use disorders was 91%. Representatives from each facility responded to a 37-item questionnaire, via secure web-based survey, a paper questionnaire by mail, or a telephone interview. Further details about the survey design, data collection, and the dataset are available through SAMHSA [33].

Our outcome of interest was whether OTPs offer a substance use treatment program or group specifically tailored for CJ-involved clients (yes/no). Importantly, OTPs in N-SSATS are not based in carceral settings, but rather serve the general population, which includes those who may be on probation, parole, or under the supervision of some other community-based court system. This variable was derived from an item in the questionnaire that asked, “For which client categories does this facility at this location offer a substance use treatment program or group specifically tailored for clients in that category? If this facility treats clients in any of these categories but does not have a specifically tailored program or group for them, do not mark the box for that category.” Our outcome was defined by OTPs that marked the only response option that addressed CJ, which was, “Criminal justice clients (other than DUI/DWI)”. Examples of other groups that could be selected include adolescents, seniors or older adults, active duty military, and clients who have experienced trauma. There is no additional information available in N-SSATS that describes each tailored service in more detail.

We defined whether an OTP was in a Medicaid expansion state using a report by the Henry J. Kaiser Family Foundation [34]. To match with the most recent N-SSATS data available (2019), we defined Medicaid expansion as any state that had adopted expansions by the end of 2019, by which time sixteen states had not expanded Medicaid under the Affordable Care Act (AL, FL, GA, ID, KS, MO, MS, NC, NE, OK, SC SD, TN TX, UT, and WI).

Based on a priori understanding of CJ, health, and Medicaid systems, both state-level overdose and community supervision rates were included in our analyses because they are related to both our dependent and independent variables. Specifically, we included state-level, age-adjusted opioid overdose death rates (per 100,000 state residents) as a confounder in our predictive model. Overdose death rates were published by the Henry J. Kaiser Family Foundation and based on publicly available data maintained by the Center for Disease Control and Prevention’s WONDER database of deaths in the US [35]. We also included the state-level community supervision rate (per 100,000 state residents) as a confounder, which was sourced form a report by the Bureau of Justice Statistics [36].

### Analysis

We used SAS Software (v9.4) [37] for all analyses. We used logistic regression to model the association between OTPs offering services tailored specifically to CJ-involved clients (outcome) and state Medicaid expansion status (predictor), adjusted for state-level opioid overdose and incarceration rates. Predicted probabilities for OTPs were calculated at the state level using the PREDICTED function within the LOGISTIC procedure in SAS. We plotted the predicted probability estimates by state and color-coded expansion decisions to visualize probability differences between groups.

## Results

A minority of OTPs offered programs or groups specifically tailored towards CJ-involved clients (26%, *n* = 443), and the majority of OTPs were located in Medicaid expansion states (73%, *n* = 1238). The mean opioid overdose rate was 17.3 per 100,000 (standard deviation [SD] = 8.99), with the lowest and highest overdose rates identified in Nebraska (3.3 per 100,000) and West Virginia (42.4 per 100,000), respectively. The mean community supervision rate was 1767.4 per 100,000 (SD = 986.0), with the lowest and highest community supervision rates identified in New Hampshire (570.0 per 100,000) and Georgia (5369.0 per 100,000), respectively.

**Fig. 1:**
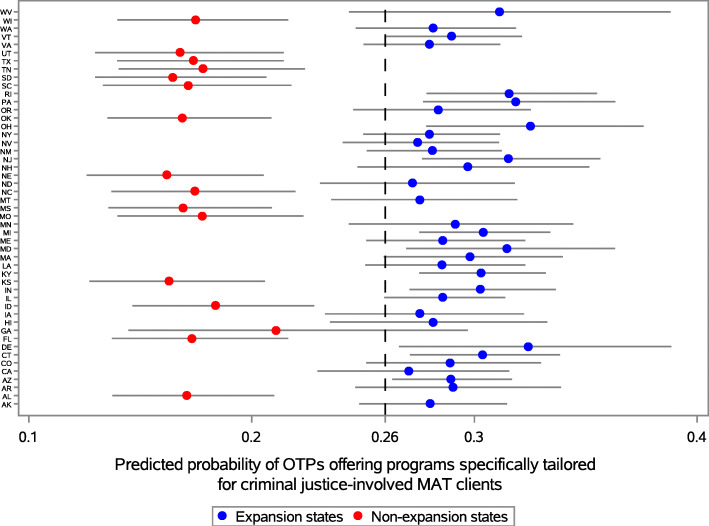
Predicted probability of OTPs offering services specifically tailored to criminal justice-involved patients, by state and Medicaid expansion status (N-SSATS; 2019; *n* = 1679 OTPs). *Note: National predicted probability = 0.26*

Holding state opioid overdose and community supervision rates constant, the odds of offering CJ-specific programs were significantly higher within OTPs in expansion states, compared to OTPs in non-expansion states (adjusted odds ratio = 1.90, 95% confidence interval = 1.41–2.57, *p* < 0.0001). We identified no statistically significant association between our confounders (state-level overdose and community supervision rates) and our outcome. The national predicted probability of OTPs offering tailored services to CJ-involved clients was 0.26; notably, point estimates for every expansion state were above the national estimate and point estimates for every non-expansion state were below the national estimate (Fig. 1).

## Discussion

Our study demonstrates that Medicaid expansion is clearly and positively associated with OTPs offering services and programs that are specifically tailored to CJ-involved MAT clients. The probability of offering tailored services to CJ populations was higher than the national average in all OTPs in expansion states, and lower than the national average in all OTPs in non-expansion states. This finding is in line with previous work reporting that CJ populations have benefited widely from expanded Medicaid services [38–41]. Generally, treatment and health outcomes are better in services that are uniquely tailored to the needs of a particular group [42–45]. In the context of programs for CJ-involved MAT clients, tailored services could include assistance with health and legal system navigation, overdose prevention in the weeks immediately following release, and assistance integrating back into family and community life while adhering to MAT regimens.

Medicaid expansion is an important factor in identifying state characteristics associated with OTP services. However, Khatri and colleagues [46] recently identified that the use of MAT was already higher in expansion states prior to the implementation of the ACA as far back as 2008. Thus, other factors within expansion states may also help explain their better outcomes. At the same time, the role of Medicaid expansion in bolstering MAT availability for CJ-involved clients cannot be discounted. In the same report, the authors identified a 165% increase in the use of MAT among clients referred to treatment by a CJ agency, while no change in CJ-referred MAT was identified among non-expansion states. Our findings are aligned with those of Khatri and colleagues in that Medicaid expansion is an important driver of MAT access for CJ-involved OUD clients.

OTPs in expansion states may have access to more funding and resources that facilitate the implementation of additional tailored services. Our results also complement previous findings that OTPs in Medicaid expansion states are more likely than OTPs in non-expansion states to offer integrated and comprehensive mental health services [47]. Our analysis suggests that disadvantaged groups in need of MAT, especially those groups impacted by CJ-involvement, may have more opportunities to access a broader range of services in expansion states. Promoting the adoption of Medicaid expansion in states that have not implemented it, as well as strengthening existing programs in expansion states, are potential ways to improve health outcomes among CJ-involved individuals who often have multiple intersecting health exposures [48–51]. Research from San Francisco’s Transitions Clinic Network has demonstrated that tailoring substance use treatment services to the unique needs of CJ-involved individuals is effective in improving health outcomes and reducing recidivism [52]. Similarly, tailored services within OTPs may improve treatment engagement, treatment longevity, and MAT completion, all of which are related to positive outcomes like reduced opioid use and fewer overdoses [3, 53].

Some researchers have recently conceptualized what CJ-tailored services may look like on a broader scale. For example, Brinkley-Rubinstein and colleagues [21] recently proposed the Criminal Justice Continuum of Care for Opioid Users at Risk of Overdose. Their continuum of care could be adapted for use specifically in OTPs, with emphasis on collaborating across the health, law enforcement, and community sectors and the provision of naloxone for overdose prevention. Wrap-around services to assist with employment and housing have long been goals of supporting individuals with drug use disorders and those involved in the CJ system, but the majority of OTPs do not offer supportive services like childcare, transportation, or housing assistance [54]. A public health strategy for dealing with the opioid epidemic includes increasing resource allocation for supportive services [55]. OTPs are positioned well to advance this public health approach by tailoring services to improve MAT outcomes for clients involved in the criminal justice system. Broader state and national health policies likely affect the ability of OTPs to create and sustain such tailored services.

### Limitations

While this is the first study to identify national trends in tailored CJ services within OTPs, our definition of programs and services specifically tailored to CJ-involved MAT clients is limited. N-SSATS does not provide detailed information about specific characteristics of tailored programs, nor does it provide demographic characteristics of individual treatment clients. A fuller and more in-depth understanding of tailoring services to vulnerable groups is needed in order to better inform health policy and to optimize clinical MAT practices. Future research could fill this gap by identifying specific characteristics of programs tailored for CJ-involved clients that are the most effective in engaging these clients and improving their outcomes.

Our study only utilized 1 year of N-SSATS data. As a cross-sectional analysis of 2019 data, we are limited to demonstrating associations and are unable to identify a causal pathway between Medicaid expansion and changes in OTP services. Future research may address temporal changes in OTP services and differences by Medicaid expansion status, especially as several states have yet to opt into Medicaid expansion and the opioid and mass incarceration crises are not abating.

## Conclusion

Individuals involved in the CJ system make up a sizable portion of the overall MAT clientele in OTPs. OTPs in Medicaid expansion states are significantly more likely than OTPs in non-expansion states to offer programs specifically tailored to the needs of CJ-involved clients, reiterating the role of Medicaid in promoting the adoption of comprehensive services that provide more support to CJ-involved populations. However, tailored services remained low across all 50 states, with no state having more than 40% of its OTPs offering such programs. While promoting the adoption of Medicaid expansion is critical, this research also point to the need to continually strengthen MAT services in all states, including expansion states, with a focus on developing new services and programs that specifically target populations with the highest need.

## Data Availability

All data used in this study is free, unrestricted, and publicly available online. The National Survey of Substance Abuse Treatment Services is available through SAMHSA: https://wwwdasis.samhsa.gov/dasis2/nssats.htm State-level, age-adjusted opioid overdose death rates are available through the Kaiser Family Foundation: https://www.kff.org/other/state-indicator/opioid-overdose-death-rates/?currentTimeframe=0&sortModel=%7B%22colId%22:%22Location%22,%22sort%22:%22asc%22%7D State-level prison incarceration rates are available through the Sentencing Project: https://www.sentencingproject.org/the-facts/#detail?state1Option=U.S.%20Total&state2Option=0

## Abbreviations

OTP: Opioid treatment program
OUD: Opioid use disorder
MAT: Medication-assisted treatment
CJ: Criminal justice
N-SSATS: National Survey of Substance Abuse Treatment Services
SAMHSA: Substance Abuse and Mental Health Services Administration
